# Patent blue and air as an alternative for resection of nonpalpable breast lesions: a case series: Azul patente e ar como uma alternativa para a ressecção de lesões não palpáveis de mama: série de casos

**DOI:** 10.1590/1516-3180.2014.1321559

**Published:** 2014-02-01

**Authors:** Sabas Carlos Vieira, Viviane Carvalho Alves, Tayná Cristinne Barros de Oliveira, Jacira Oliveira Ibiapina, Emmyle Cristyne Alves Soares, Marcus Luciano Lopes de Paiva Crisanto

**Affiliations:** I PhD. Adjunct Professor of Oncology, Department of General Practice, Universidade Federal do Piauí, Teresina, Brazil; II Medical Student, Universidade Federal do Piauí, Teresina, Brazil

**Keywords:** Breast neoplasms, Coloring agents, Biopsy, Mammography, Early detection of cancer, Neoplasias da mama, Corantes, Biópsia, Mamografia, Detecção precoce de câncer

## Abstract

**CONTEXT AND OBJECTIVE:**

Use of mammography for breast cancer screening has resulted in a significantly increased number of patients with nonpalpable radiological findings that need histopathological study for better management. The present study evaluated an alternative to excision of nonpalpable breast lesions, using injection of patent blue (CAS 3536-49-0) dye and air.

**DESIGN AND SETTING:**

Cohort study of 64 consecutive patients at a private clinic in the city of Teresina (Piauí), between January 2009 and December 2010.

**METHODS:**

: The patients had received mammographic diagnoses of nonpalpable breast lesions classified as BI-RADS 3, 4 and 5, with indication of histopathological study. They underwent stereotaxy and/or ultrasound-guided injection of patent blue, for marking and subsequent excision of the lesion.

**RESULTS:**

: The patients' mean age was 47.7 years. Nodes accounted for 53.1% of the breast abnormalities; microcalcifications, 37.5%; and complex cysts, 9.4%. In 89.1% of cases, the lesions were BI-RADS 4; 7.8% were BI-RADS 5 and 3.1% were BI-RADS 3. The histopathological findings were benign in 70.3% of the cases; atypical hyperplasia, 9.4%; and malignant, 20.3%. Among the malignant cases, 53.8% were carcinoma *in situ* and 46.2%, invasive carcinoma. The percentage of malignancy was 0% in BI-RADS 3 lesions; 14.3% in BI-RADS 4 and 100% in BI-RADS 5. In the cases of malignancy, the margins were clear in 92.3%. Reoperation to widen the margins was required in one patient.

**CONCLUSION:**

Excision of nonpalpable breast lesions marked with patent blue and air was possible in all cases.

## Abstract (pt)

**CONTEXTO E OBJETIVOS:**

A utilização da mamografia no rastreamento para o câncer de mama tem apresentado significativo aumento do número de pacientes com achados radiológicos impalpáveis que, para melhor orientação terapêutica, necessitam de estudo histopatológico. O presente estudo avaliou uma alternativa à ressecção de lesões impalpáveis de mama utilizando injeção de corante azul patente e ar.

**TIPO DE ESTUDO E LOCAL:**

Coorte de 64 casos consecutivos de uma clínica particular da cidade Teresina (PI), no período de janeiro de 2009 a dezembro de 2010.

**MÉTODOS:**

As pacientes receberam o diagnóstico mamográfico de lesões impalpáveis de mama classes BI-RADS 3, 4 e 5 com indicação para estudo histopatológico. Elas foram submetidas à injeção de azul patente, orientada por estereotaxia e/ou ultrassonografia para marcação e posterior ressecção da lesão.

**RESULTADOS:**

A média de idade das pacientes foi 47,7 anos. Os nódulos representaram 53,1% das anormalidades; as microcalcificações, 37,5% e os cistos complexos, 9,4%. Em 89,1% dos casos, as lesões eram BI-RADS 4; 7,8%, BI-RADS 5 e 3,1%, BI-RADS 3. Os achados histopatológicos foram benignos em 70,3% dos casos; com hiperplasia atípica em 9,4% e malignos em 20,3%. Dos casos de malignidade, 53,8% eram carcinoma *in situ* e 46,2%, carcinoma invasor. O percentual de câncer foi 0% nas lesões BI-RADS 3;14,3% nas BI-RADS 4 e 100% nas BI-RADS 5. Nos casos de neoplasia maligna, as margens estavam livres em 92,3%, sendo necessária reoperação para ampliação de margens em uma paciente.

**CONCLUSÃO:**

A ressecção de lesões impalpáveis de mama marcadas com azul patente e ar foi possível em todos os casos.

## INTRODUCTION

The dissemination of breast cancer screening associated with better imaging techniques has resulted in increased incidence of nonpalpable breast lesions, as classified according to the Breast Imaging Reporting and Data System (BI-RADS), published by the American College of Radiology (ACR) and recommended by the Brazilian College of Radiology (CBR).^1^

The initial approach is to perform core biopsy or complete excision of the lesion by means of mammotomy. In cases of inconclusive biopsy or in the presence of carcinoma, surgery is indicated. In nonpalpable lesions, surgical biopsy should be preceded by preoperative ultrasound-guided or mammography-guided marking, (freehand, biplanar or stereotactic).^2^ The exact preoperative site of the lesion is a determining factor for whether high rates of total resection are achieved, thereby decreasing the need for re-excision.

Currently, the most widely used methods involve techniques with radioactive material (radioguided occult lesion localization, ROLL), metal wire, activated charcoal or dyes, such as patent blue,^3^ methylene blue^4^ and indocyanine green.^5^ Nowadays, the majority of breast surgery services use ROLL as the standard procedure. In a literature review conducted by the authors of the present study, only nine studies used dye for localization of nonpalpable breast lesions.^3^ ^-^ ^11^ Among these studies, two used a dye in association with ROLL^6^ ^,^ ^7^ and one used a dye in association with a metal wire.^8^ Of these nine, only two studies were Brazilian.^3^ ^,^ ^10^ The aim of the present study was to evaluate the resection of nonpalpable breast lesions stained with patent blue dye and air.

## METHODS

This was a retrospective cohort study. The medical files of 64 patients seen at a private breast disorder clinic in the city of Teresina (PI) between January 2009 and December 2010 were analyzed after gaining approval from the Ethics Committee of Universidade Federal do Piauн. These patients had received imaging diagnoses of nonpalpable breast lesions classified as BI-RADS 3, 4 and 5 and an indication for histopathological study. The imaging diagnosis was performed by means of mammography and/or breast ultrasonography.

On the day of the surgery, the lesions were marked with patent blue dye as close as possible to the time scheduled for the surgical procedure. The interval between dye injection and the surgical procedure ranged from 21 to 320 minutes, with a mean time of 156 minutes.

**Figure 1:**
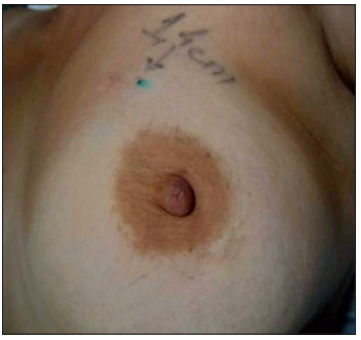
Marking of puncture site with the shortest route.

Initially, 2% lidocaine chloride was infiltrated at the puncture site where the dye would be injected. Subsequently, 0.2 ml of patent blue dye and 0.4 ml of air were injected by means of a syringe that was stereotaxy-guided and ultrasound-guided or only ultrasound-guided. The purpose of dye injection was to facilitate lesion localization by the surgeon, using ultrasound. After the end of the procedure, ultrasonography was performed to mark the skin location with the shortest tissue route for excision of the lesion. This site was used to guide surgical incision (Figure 1).

**Figure 2:**
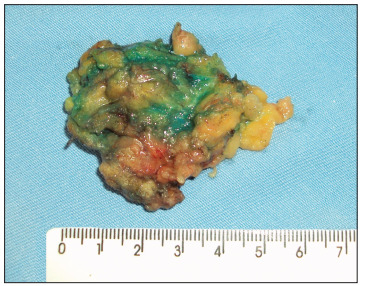
Excised blue node.

The surgical procedure was performed under local anesthesia and sedation. Antibiotic prophylaxis was not administered. A perpendicular incision was made in the subcutaneous tissue and breast parenchyma until the area marked in blue had been located. The node marked in blue was then excised (Figure 2) and subjected to histopathological analysis. Specimens with microcalcifications were previously radiographed in order to confirm that complete excision of the lesion had been achieved. Hemostasis was checked. The incision was subsequently closed using a fine skin suture and compression dressing was applied. No drainage tube was used. 

## RESULTS

The patients' mean age was 47.7 ± 7.5 years. In 89.1% of the cases, the lesions were classified as BI-RADS 4, while 3.1% were BI-RADS 3 and 7.8% were BI-RADS 5 (Table 1). Nodes accounted for 53.1% of the abnormalities, microcalcifications for 37.5% and complex cysts for 9.4% (Table 2).

**Table 1:**
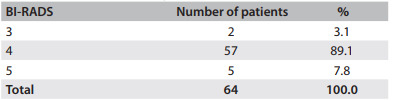
Patients according to BI-RADS classification

BI-RADS = Breast Imaging Reporting and Data System

**Table 2:**
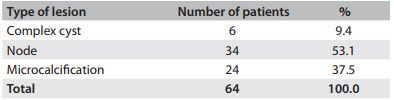
Types of lesions according to radiological characteristics

Twenty-five patients underwent biopsy prior to lesion excision. Of these, 7 cases were malignant, 14 were benign, 2 consisted of atypical hyperplasia and 2 were inconclusive. We considered that the lesions classified as BI-RADS 4C or 5, in which biopsies did not reveal malignancy due to possible sampling error, were inconclusive. Out of the 14 lesions previously classified as benign, 12 (87.5%) were benign, one (6.25%) showed atypical hyperplasia and one (6.25%) had a final result of malignancy after excision. Neither of the two cases of atypical hyperplasia was confirmed as such: one (50%) was revealed to be malignant, while the other (50%) proved to be benign. All seven cases previously diagnosed as malignant was confirmed as such. The two cases with previously inconclusive biopsies proved to be benign after excision (Figure 1).

According to the pathological analyses on the excised specimens, 70.3% of the lesions were benign and 9.4% were atypical hyperplasia. Among the benign lesions, fibroadenoma predominated (35.6%). In 20.3% of the cases, the final histopathological diagnosis was breast cancer. Of these, 10.9% were carcinoma *in situ* and 9.4% were invasive carcinoma (Table 3). All of these cases (both the carcinoma in situ and invasive carcinoma cases) consisted of ductal carcinoma. One patient was re-excised to widen the margins of excision. None of the BI-RADS 3 lesions were malignant. Among the BI-RADS 4 lesions, the percentage of cancer was 14.3% and among the BI-RADS 5 lesions, 100% (Table 4).

**Table 3:**
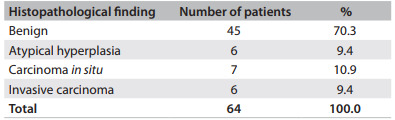
Patients according to histopathological study on resected lesions

**Table 4:**
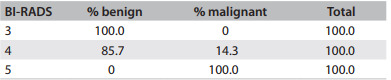
Percentage of benign and malignant lesions, according to BI-RADS classification

BI-RADS = Breast Imaging Reporting and Data System.

Five patients with a malignant biopsy prior to resection underwent sentinel lymph node biopsy. None of these cases exhibited any compromised tissue.

## DISCUSSION

The development of mammographic techniques, as well as increased awareness about and access to breast cancer treatment has enabled earlier diagnosis and has led to higher incidence of nonpalpable breast lesions. Lesion excision is indicated when there is a previous diagnosis of malignancy (BI-RADS 6), presence of a suspicious lesion (BI-RADS 4) or a lesion highly suspected of malignancy (BI-RADS 5), or presence of lesions that are probably benign (BI-RADS 3) with an indication for surgical biopsy.^10^

In the present study, the majority (89.1%) of the patients belonged to BI-RADS category 4 (Table 1). Two BI-RADS 3 lesions were excised: one because there was a history of breast cancer in a first-degree relative and another because there was growth of a palpable and a nonpalpable node. In this case, despite the benign characteristics seen on imaging examinations, the decision to excise the lesion was made in conjunction with the patient. Several methods have been developed for surgical localization of nonpalpable breast lesions, such as metal wire, ROLL and dyes.

Metal wire-guided excisional biopsy is a safe and accurate method that has been widely adopted. However, displacement, folding or breakage of localization wires may occur. When the wire is sectioned during surgery, part may remain in the breast parenchyma, which may have the implication of legal actions brought by the patient. If the procedure is not started in the incision made at the skin puncture site for placement of the metal wire, it may be difficult to locate its tip. If an incision is made at the puncture site, the procedure will be more invasive and traumatic. Furthermore, the wire is relatively expensive.^4^

ROLL was proposed as the best alternative, compared with the standard method of wire-guided excision of nonpalpable breast lesions. A small dose (3.7 MBq) of albumin labeled with ^99m^Tc is injected by means of ultrasound or stereotactic imaging. During surgery, the lesion is located using a gamma ray probe.^6^ The main disadvantage of this technique consists of difficulty in establishing the depth of the breast parenchymal lesion, since the probe cannot distinguish between the depths of the lesion, which may give rise to wider excision of the breast segment than desired.^12^ This difficulty was found to be overcome when a dye is used in association with ROLL for marking nonpalpable lesions.^7^ In that study, 157 patients with nonpalpable lesions were evaluated. Among these, marking was performed using a metal wire in 78 cases and ROLL associated with methylene blue in 79 cases. Surgery and ROLL combined with methylene blue was performed in a shorter time and achieved a clearer surgical margin and a smaller specimen size. In addition, there was a lower rate of re-excision and the size of the skin incision was smaller than with wire-guided excision.^7^ However, with regard to marking, use of dye alone is sufficient to completely excise the lesion, as demonstrated in our current study.

Another disadvantage of using ROLL is that an additional professional is required in the team (the nuclear physician), thus raising the cost of the procedure, since it includes performing breast scintigraphy. According to research carried out by the present authors in private clinics in the city of Teresina (capital of the state of Piauн), ROLL surpasses the costs of excision with dyes by about 75%.

The effectiveness of dye used alone was also demonstrated in another study, in which 57 lesions of 51 patients were marked with 0.4-0.7 ml of methylene blue, with ultrasound guiding, 20 to 180 minutes before the surgical procedure.^9^ All the lesions were successfully excised. Adequate localization was possible in the cases of 56 lesions. In a single patient, the lesion was not found because the dye had been absorbed before the beginning of the procedure. In that case, the lesion was excised using intraoperative ultrasound. The authors attributed this failure to the interval of 100 minutes between dye injection and the beginning of surgery, and suggested that the surgery should be started as soon as possible after dye injection. The procedure involving dye injection was reported as painful by 5.3% of the patients, tolerably painful by 28% and painless or mildly uncomfortable by 66.7%. No allergic reactions were observed.^9^

In a study by Prudкncio et al., patent blue dye was used in preoperative marking of 285 patients with nonpalpable breast lesions. In 153 patients, the marking was guided by ultrasound and, in 132 patients, mammography was used. Marking was performed between 30 and 180 minutes before the surgical procedure. The marked area was identified during the intraoperative period and was successfully excised in all cases (100%).^10^

The main advantage of marking with a dye is that it enables lesion removal under direct viewing of the blue area. In the past, after dye injection, the route of the puncture site was marked during needle removal. The incision was made along the marked track. If the puncture site was situated far from the lesion, wider incisions and trauma were inevitable, thereby compromising the cosmetic results from the procedure.^4^ A small amount of air between the plunger and the dye is currently used, which facilitates lesion localization by means of ultrasound, thus marking the site closest to the lesion to be excised. The puncture path is not impregnated with dye, which gives rise to less tissue trauma. The excision is only made when the marked blue area is viewed. Thus, there is a smaller area of excised breast parenchyma, which improves the cosmetic results.^3^ One initial concern with this method was the dissemination of dye, which could make the technique difficult if surgery was not performed promptly after injection.

One important advantage of patent blue is its capacity to diffuse to adjacent tissues. Its capacity to diffuse is intermediate, in comparison with methylene blue, which diffuses greatly, and charcoal, which does not diffuse. Since it does not require the use of nuclear medicine and special metal wires, the cost of the procedure is also lower. As shown in this case series, the time that elapsed between dye injection and performing the surgical procedure was 163 minutes on average, ranging from 21 to 320 minutes. In all cases, the dye was restricted to the area of nonpalpable lesion, thus not compromising the excision. In patients with invasive carcinoma and carcinoma *in situ*, the margins were not clear in only one patient, who needed to be re-excised to widen the margins of excision. 

In order to introduce an alternative technique and determine its applicability for surgical removal of nonpalpable breast lesions, Aydogan et al. performed occult lesion localization guided with indocyanine green dye, in a case report. Lesion localization was performed in two patients before surgery under ultrasonographic control by injecting indocyanine green into the lesion and its subcutaneous tissue projection. During surgery, the site of the skin incision and the resection margins were identified by observing the area of indocyanine-derived fluorescence under the guidance of a near-infrared-sensitive camera. In both cases, the breast lesion was correctly localized, and the area of fluorescence corresponded well to the site of the lesions. On histopathological examination, the surgical margins were found to be clear. The authors concluded that indocyanine green is an excellent dye for localization of nonpalpable breast lesions.^5^

Another argument against the use of dyes is the possibility of allergic events. In the present case series, there was no occurrence of allergic reaction. In the literature, the incidence of allergic phenomena seen through using patent blue dye has been 0.06 to 2.7%, with a mean value of 0.71%,^13^ particularly in surgery for investigation of sentinel lymph nodes in which a higher volume is used, usually 2 to 4 ml. When nonpalpable lesions are marked, only 0.2 ml is used. One precautionary measure is to avoid performing this procedure in patients with a significant history of allergy, such as severe hives and angioedema.

## CONCLUSION

Excision of nonpalpable breast lesions marked with patent blue dye and air was possible in all lesions. In the cases of malignancy, there were clear margins in 92.3%, while re-excision to widen the margins of excision was required in one patient.
